# New data from the first discovered paleoparadoxiid (Desmostylia) specimen shed light into the morphological variation of the genus *Neoparadoxia*

**DOI:** 10.1038/s41598-022-18295-5

**Published:** 2022-08-21

**Authors:** Kumiko Matsui, Ana M. Valenzuela-Toro, Nicholas D. Pyenson

**Affiliations:** 1 Department of Paleobiology, National Museum of Natural History, Smithsonian Institution, 10th Street Northwest & Constitution Ave. NW, Washington, DC 20560 USA; 2 The Kyushu University Museum, Kyushu University, Fukuoka, Japan; 3 Department of Ecology and Evolutionary Biology, University of California Santa Cruz, Coastal Biology, Santa Cruz, CA USA; 4 Department of Paleontology and Geology, Burke Museum of Natural History and Culture, Seattle, WA USA

**Keywords:** Palaeontology, Palaeoecology

## Abstract

Desmostylia is an extinct clade of marine mammals with two major sub-clades, Desmostylidae and Paleoparadoxiidae, known from Oligocene to Miocene strata of the North Pacific coastline. Within Paleoparadoxiidae, three genera have been identified: *Archaeoparadoxia*, *Paleoparadoxia*, and *Neoparadoxia*. The latter taxon is the geochronologically youngest palaeoparadoxiid and *Neoparadoxia* is characterized by a comparatively larger body size, although it is known only from a few specimens within a short temporal and geographic range. Here we report the discovery of an isolated tooth, which we identify as *Neoparadoxia* cf. *N. cecilialina*, constituting only the second individual specimen of *Neoparadoxia* with preserved dentition yet reported. This specimen was collected near Corona, California, USA, and we attribute it to the “Topanga” Formation, extending the geographic range of this taxon in Southern California. While the exact geographic locality was not recorded when it was collected in 1913, we establish two potential localities based on associated hand-written museum label and new stratigraphic information. Although initially identified as *Desmostylus hesperus*, this specimen of *Neoparadoxia* was collected 10 years before the first named paleoparadoxiid from Japan. We expect that description of more complete desmostylian material from elsewhere in Southern California will clarify the taxonomic richness and paleoecological role of this clade in Cenozoic marine mammal assemblages.

## Introduction

Desmostylia is an enigmatic extinct group of quadrupedal and herbivorous marine mammals known from Oligocene and the Miocene marine strata of both coasts of the North Pacific Ocean (e.g.^1–3^). Within Desmostylia, Paleoparadoxiidae is a monophyletic family (e.g.^4,5^), comprising four species distributed in three genera (namely *Archaeoparadoxia*, *Paleoparadoxia*, and *Neoparadoxia*^3,5^). Fossil remains of paleoparadoxiids have been found in marine deposits ranging in age from the latest Oligocene (Chattian) to the earliest late Miocene (Tortonian) of the North Pacific coasts of Japan and the United States (24 to 10 Ma^3,6,7^).

Paleoparadoxiidae has an extensive and complicated taxonomic history, characterized by several changes in its nomenclature. This family was founded in 1923^8,9^ with the discovery of two isolated teeth (likely belonging to two different individuals; see more below) from middle-late Miocene (late Serravallian to early Tortonian) marine strata of the Tsurushi Formation, on Sado Island, Niigata Prefecture, Japan (see^10^ for age revisions to the Tsurushi Formation). These specimens (including a left second molar (m2) designated as the type) were originally identified as *Cornwallius tabatai*^8^; however, they were both presumed destroyed in 1945 during the Second World War, with only Tokunaga's original photos and illustrations as reference material. Subsequently, a nearly complete desmostylian skeleton was discovered in 1950, at Izumi (currently Toki City), Gifu Prefecture, Japan. This skeleton from Izumi included forelimbs, cranium, and a mandible (NMNS PV-5601) that was originally recognized as *Desmostylus* sp.^11^, but was then reidentified as belonging to *C. tabatai*^12^. Years later, when Reinhart^4^ revised desmostylian taxonomy, he established the new genus *Paleoparadoxia* and recombined *Paleoparadoxia tabatai* based on the *C. tabatai* holotype specimens (which Reinhart may not have known were lost) along with specimens from Southern California (UCMP 40862 and UCMP 32076). Later, Shikama^13^ designated the Izumi specimen (NMNS PV-5601) as the neotype of *P. tabatai*.

Clark^6^ described a second species of *Paleoparadoxia*, *P.weltoni*, based on a smaller but fully adult complete skeleton (UCMP 114285) from the earliest Miocene Schooner Gulch Formation (Aquitanian) in northern California (see^14^ for the age of the Schooner Gulch Formation). Later, Inuzuka^9^ described postcranial material (UCMP 81302) collected during the construction of the Stanford Linear Accelerator Center (now the SLAC National Accelerator Laboratory) near Menlo Park, California, as belonging *Paleoparadoxia* sp. Based on this specimen from the middle Miocene Ladera Sandstone (Langhian-Serravalian), Inuzka^9^ proposed that the lost holotype of *Cornwallius tabatai* likely corresponded to the same species represented as the so-called “Stanford specimen” (UCMP 81302) based on its molar and estimated size, yet he conducted no further comparisons.

At the same time, Inuzuka^9^ proposed yet another new species name, *Paleoparadoxia media*, for the neotype specimen of *P. tabatai* from Izumi*.* The basis for this decision stems from Inuzuka’s apparent rediscovery of one fossil tooth that probably belonged to the same individual of the original holotype specimen of *P. tabatai* from Aikawa Local Museum near the type locality, a left third molar (m3), from the Orito Formation from Sado Island (see^9,15^). Although it is possible that this second specimen belongs to the same individual as the type left m2 of *P. tabatai*, this nomenclatural decision is problematic because it is destabilizing, attributing an older and less complete specimen to the typology of *P. tabatai*. Hasegawa and Kohno^16^ petitioned the ICZN (Case 3384) to conserve the name of *P. tabatai* by fixing the Izumi specimen as the lectotype, along with the suppression of *P. media*. The ICZN^17^ declined this petition (ICZN Opinion 2232) on the basis of an invalid lectotype designation by Inuzuka^9^, which had the effect of maintaining Shikana’s^13^ designation of the Izumi specimen as the neotype, consistent with over a half century of desmostylian taxonomic work. We further recommend the suppression of *P. media*, in keeping with this practice.

Domning and Barnes^15^ performed a comprehensive morphological assessment of UCMP 81302 and identified it as a new species, *Paleoparadoxia repenningi*, until Barnes^3^ transferred this species to the new genus *Neoparadoxia*, thereby recombining the species as *N. repenningi.* In this same study, Barnes^3^ also transferred the holotype specimen of *Paleoparadoxia weltoni* (UCMP 114285) to a new monotypic genus *Archaeoparadoxia* and described a new species of *Neoparadoxia*, *N. cecilialina* based on a well preserved and complete skeleton including the cranium, mandible, and teeth (LACM 150150) from the early late Miocene Monterey Formation (Tortonian), in Southern California (see below).

*Neoparadoxia cecilialina* is the geochronologically youngest desmostylian taxon and ranks as the largest paleoparadoxiid, reaching an estimated standard length of 2.73 m as an adult^3^, and possessed pectoral limb dimensions 1.5 to 2 times the size of other paleoparadoxiids. Nonetheless, this species is known by a single specimen (its holotype), which makes it difficult to assess intra- and interspecific variation of paleoparadoxiid cranial and postcranial morphology and, ultimately, testing any ecomorphological hypotheses among co-occurring desmostylians.

**Figure 1:**
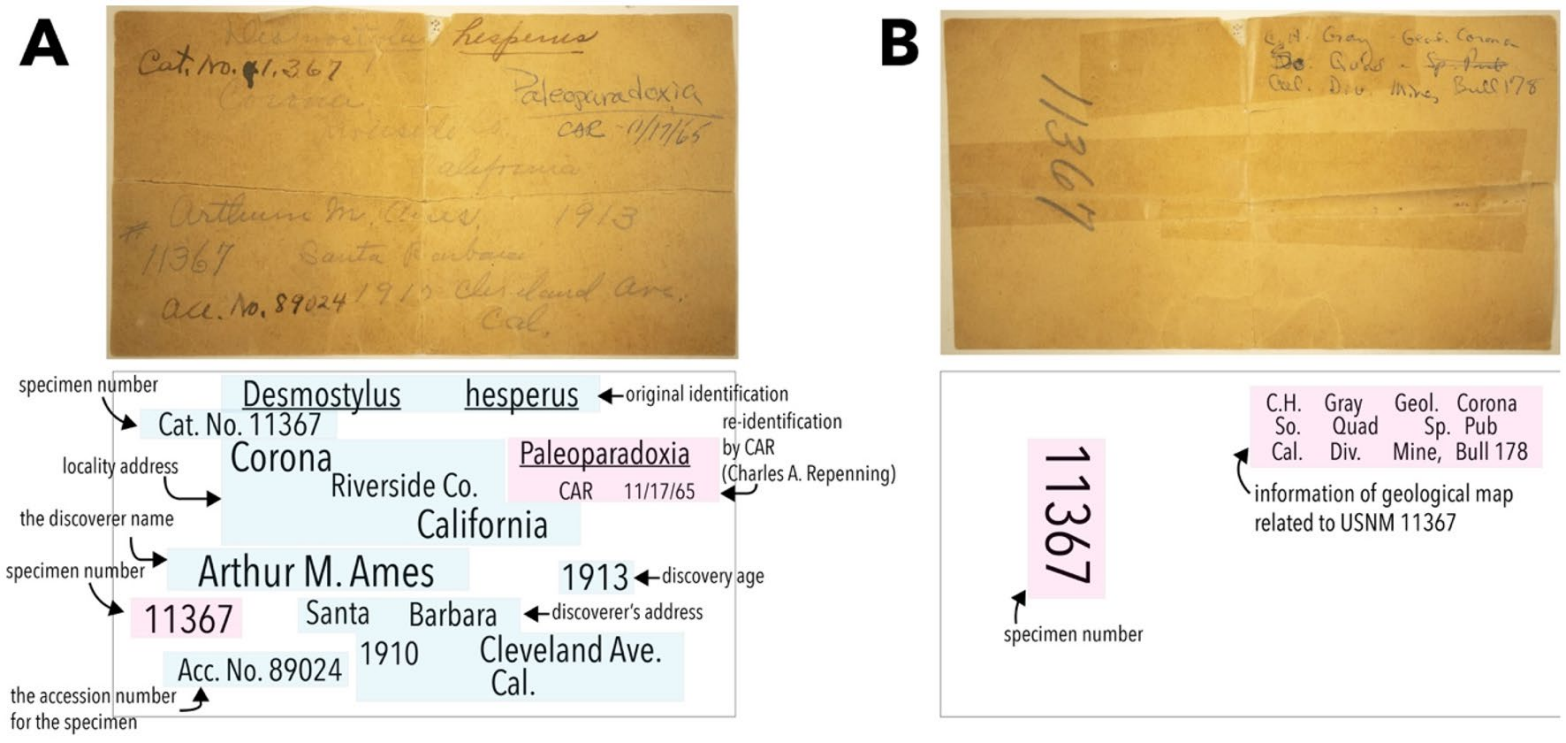
The original label of USNM PAL V 11367. (**A**) A front side; (**B**) a backside. Blue masked areas are old descriptions, and pink areas were presumably added later. In addition, “ACC (accession number) No. 89024” was also written on the label, but we could not identify its meaning. This label is also housed in the National Museum of Natural History, Smithsonian Institution (Washington DC, USA).

In the summer of 2021, one of the authors (AVT) found a desmostylian specimen (USNM PAL V 11367) in the Department of Paleobiology collections of the National Museum of Natural History, Smithsonian Institution. USNM PAL V 11367 corresponds to a complete lower molar with diagnostic features of Paleoparadoxiidae (listed in Systematics) and comparable in morphology to *Neoparadoxia cecilialina*. The original museum label with this specimen (Fig. 1) indicates that it was originally collected in Corona, Riverside County, California, USA, in 1913, 10 years before the discovery of the first Paleoparadoxiidae remains (the lost holotype of *P. tabatai*)*,* and 51 years before the discovery of the *Neoparadoxia repenningi*. Thus, USNM PAL V 11367 represents the historically oldest paleoparadoxiid specimen. Finally, this finding provides new information about the morphological variation of paleoparadoxiids.

## Systematics

Desmostylia Reinhart 1953^18^.

Desmostyloidea Osborn 1905^19^ sensu Matsui and Tsuihiji 2019^5^.

Paleoparadoxiidae Reinhart 1959^4^ sensu Matsui, and Tsuihiji 2019^5^.

**Diagnosis for Paleoparadoxiidae**—Molar tooth with an extra cusp present between the hypoconulid and the protoconid aligned on its posterior side^3^; tooth enamel in occlusal view is thinner than *Cornwallius* and thicker than *Ashoroa, Behemotops,* and *Seuku*; cingulum present on the buccal side.

*Neoparadoxia* Barnes 2013^3^.

**Figure 2:**
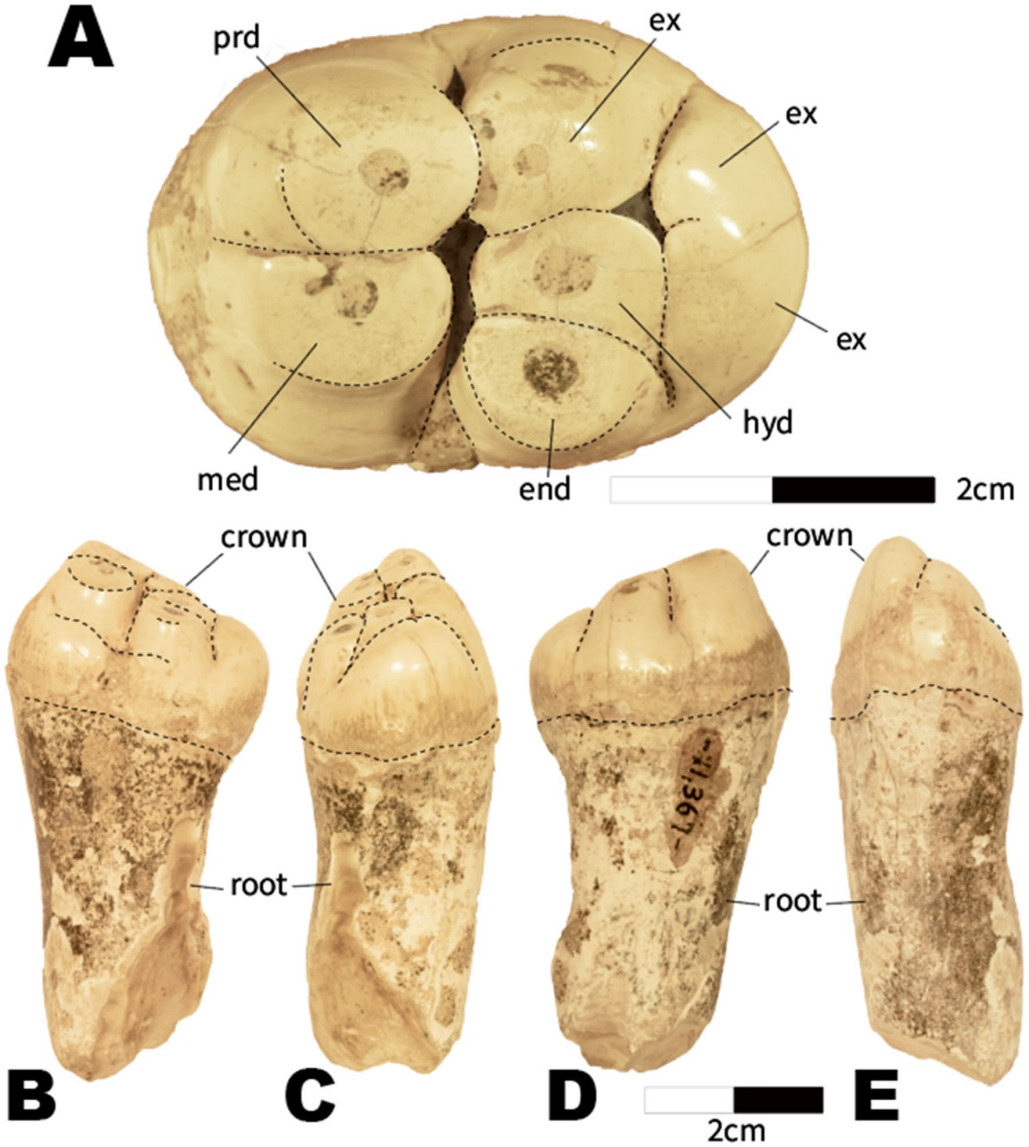
USNM PAL V 11367. (**A**) occlusal surface; (**B**) buccal view; (**C**) posterior view; (**D**) lingual side; (**E**) anterior view. All scale bars are 2 cm. prd: protoconid; med: metaconid; hyd: hypoconulid; end: entoconid; ex: extra cusp; crown: dental crown; root: dental root. This figure was created by using Adobe Photoshop and Adobe Illustrator (https://www.adobe.com/). KM took photos in this figure using the EOS M5 camera, and EF-M28mm F3.5 macro IS STM (https://canon.jp/).

**Emended diagnosis for Neoparadoxia**—Molar tooth with a higher crown than *Archaeoparadoxia* and *Paleoparadoxia*; the extra cusp (EX in Figs. 2 and 4) between the hypoconulid and protoconid is high relative to the base of the tooth crown, and enlarged compared to *Archaeoparadoxia* and *Paleoparadoxia*, reaching almost the level of the main cusps of *Archaeoparadoxia* and *Paleoparadoxia*; these latter cusps are more closely appressed to each other than in *Archaeoparadoxia* and *Paleoparadoxia*; thicker tooth enamel than *Archaeoparadoxia* and *Paleoparadoxia* but thinner than *Cornwallius*, *Ounalashkastylus*, and *Desmostylus*; extra cusps (black circles in Fig. 4) are higher than *Archaeoparadoxia* and *Paleoparadoxia*; and a weak cingulum compared to *Archaeoparadoxia* and *Paleoparadoxia*.

*Neoparadoxia* cf. *N. cecilialina.*

**Material**—USNM PAL V 11367 (Fig. 2), a right m2? with dental root. Its original label indicates the existence of associated skull material that is presumed lost.

**Formation and age**—“Topanga” Formation, Upper Burdigalian to lower Langhian (16.5–14.5 Ma)^20^. Details are provided in the discussion section.

**Figure 3:**
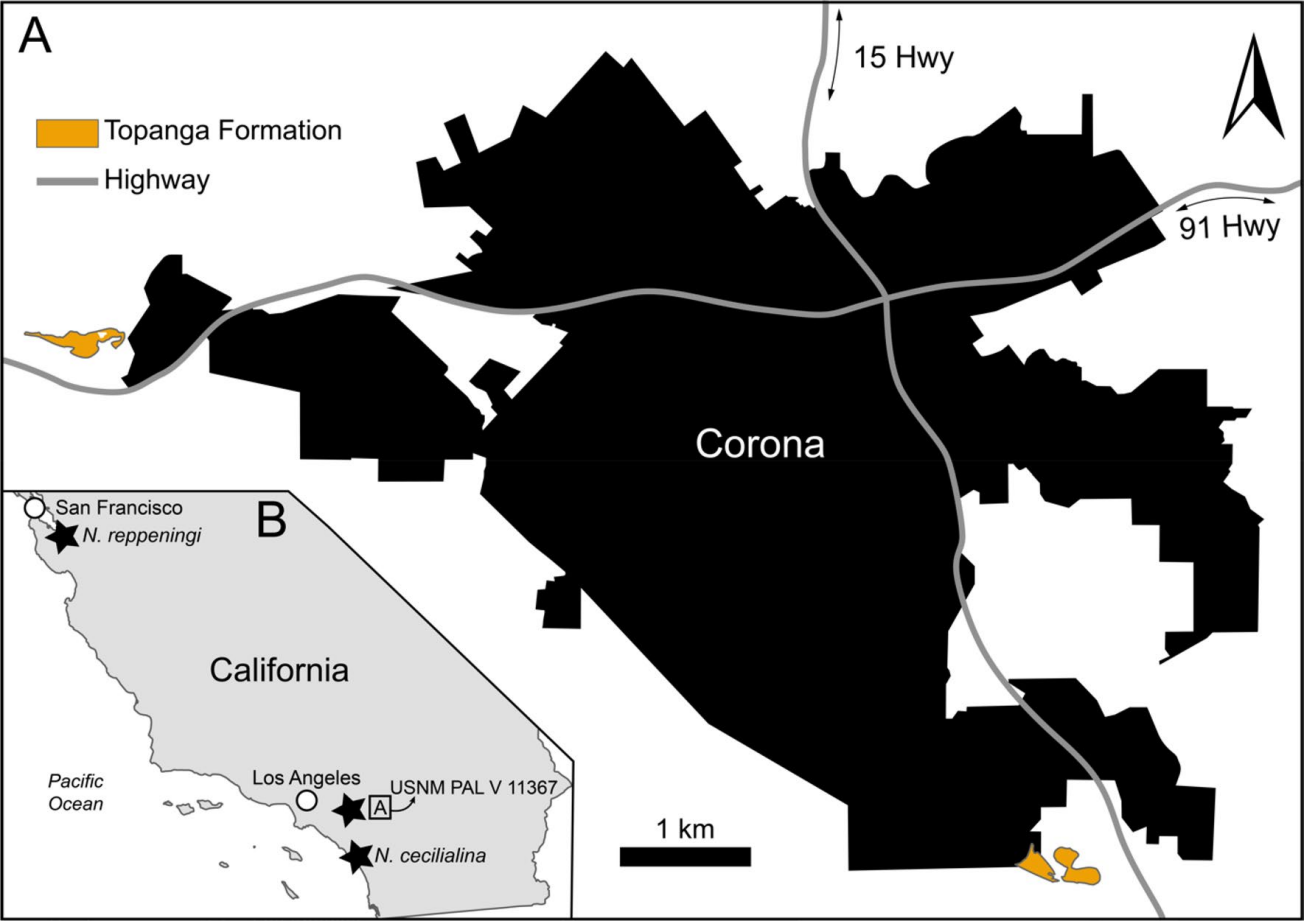
Locality map of *Neoparadoxia*. (**A**) localities of all *Neoparadoxia* previously reported and USNM PAL V 11367. This map is dawned based on geological maps published by Gray et al.^24^ and Morton et al.^34^; (**B**) potential localities of USNM PAL V 11367. Orange means distribution area of the “Topanga” Formation in Corona City. The grey line means highways. All figure was created by using Adobe Illustrator (https://www.adobe.com/).

**Potential localities**—Corona, Riverside County, California, USA. We propose two potential localities (approximately 33°52′45.7″N 117°40′49.1″W or 33°48′09.5″N 117°29′24.7″W) for USNM PAL V 11367 (Fig. 3). Details and comments are provided in the discussion section.

**Description and comparisons**—USNM PAL V 11367 has a well-worn crown with a dental root. The crown length is 32.22 mm, and its maximum width is 23.02 mm. Its crown has seven cusps. The alignment of the major cusps is consistent with m2 or m3 teeth.

USNM PAL V 11367 has a single and long root that is approximately 52.7 mm long. Only a few paleoparadoxiid molars with roots have been reported before, and little is known about their variability. Nevertheless, the type specimen of *P. tabatai*, lost to the bombing of Tokyo on 25 May 1945 by the United States during the Second World War^13,16^, had a long single root comparable to USNM PAL V 11367. Likewise, the neotype of *P. tabatai* has a single root in m3, contrasting with *N. repenningi* and *N. cecilialina*, which are characterized by having double rooted m2 and m3 alveoli, and m2, respectively (^3^; see Table S1). Similarly, *A. weltoni* has double rooted molars^6^. These observations demonstrate that molar root number in paleoparadoxiids is highly variable, indicating that this trait may not be diagnostic for genera.

**Figure 4:**
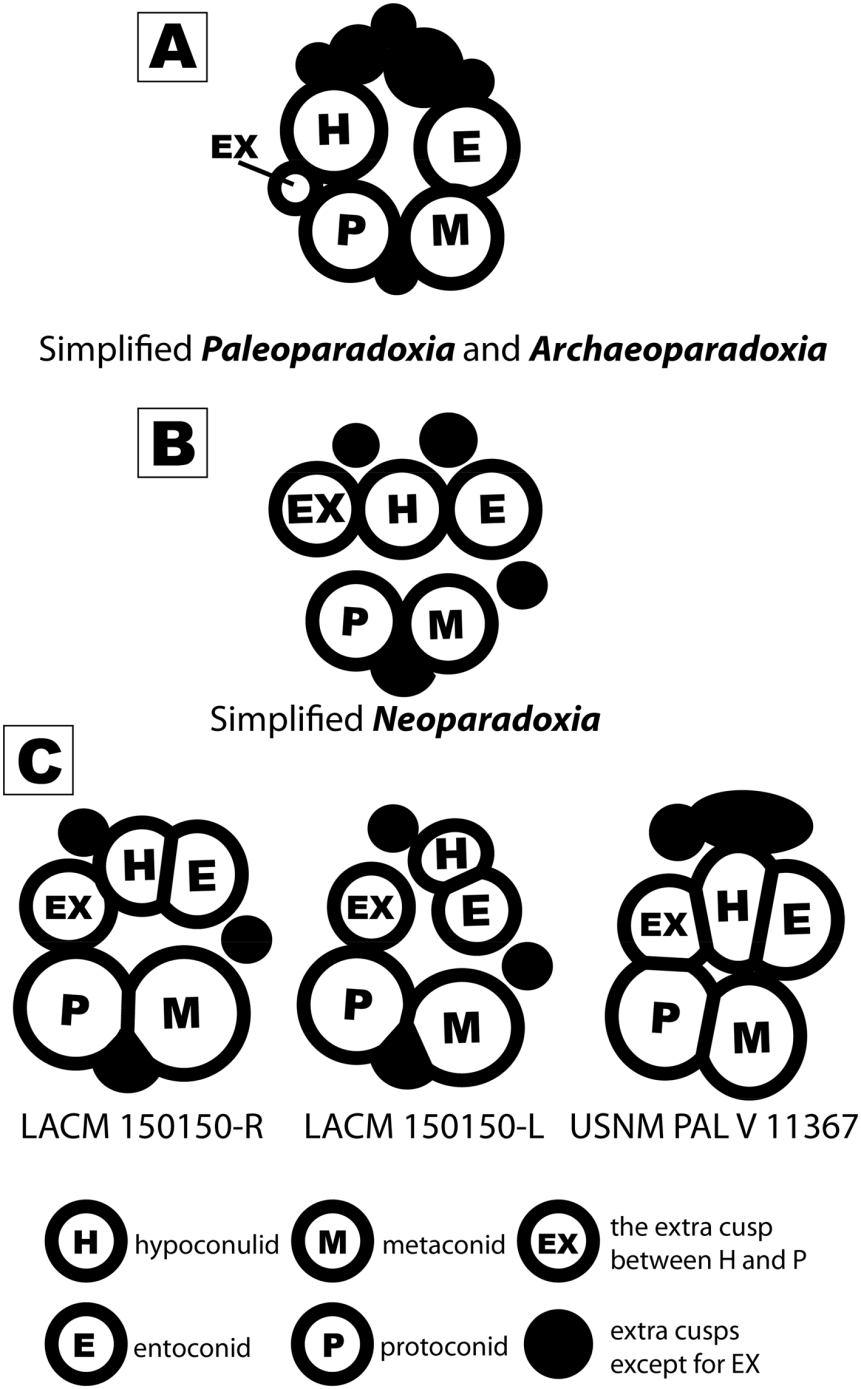
Cusps arrangements of Paleoparadoxiidae. (**A**) simplified cusps arrangement of *Paleoparadoxia* and *Archaeoparadoxia*; (**B**) simplified cusps arrangement of *Neoparadoxia*; (**C**) simple cusps arrangements of *Neoparadoxia* specimens. P: protoconid; M: metaconid; H: hypoconulid; E: entoconid; EX: the extra cusp between H and P; black circle: extra cusps except for EX. All figure was created by using Adobe Illustrator (https://www.adobe.com/).

The crown of USNM PAL V 11367 has a convex occlusal surface compared to other paleoparadoxiids but broadly is consistent with m2 of *N*. *cecilialina*. The occlusal surface is inclined in its posterobuccal side. The cingulum of USNM PAL V 11367 is located on its lingual side and is less developed than in *P*. *tabatai*. The crown in USNM PAL V 11367 is higher of its width than other paleoparadoxiids but similar to *N. cecilialina*. In total, USNM PAL V 11367 displays seven cusps, being consistent with *N. cecilialina* whose m2 also possesses seven cusps, but contrasting with *A. weltoni* and *P. tabatai*, which have five cusps on m3. Nevertheless, USNM PAL V 11367 lacks cuspules, as compared with *N. cecilialina* that shows seven major cusps with one cuspule*.* All major cusps are high, differing from all other paleoparadoxiid species with cuspules in which major cusps are variable in height. USNM PAL V 11367 has four major cusps and three extra cusps (Figs. 2 and 4). Two extra cusps on the posterior side are less developed compared to major cusps. An extra cusp exists between the protoconid and endoconid and has the same height as the major cusps. The types of *A. weltoni* and *P. tabatai* also have the same extra cusp, but these are very small compared to USNM PAL V 11367 and *N*. *cecilialina*. There is a deep groove between the protoconid-metaconid and entoconid-hypoconulid-extra cusps (dark masked area of Fig. 2). This groove is narrower and deeper than other paleoparadoxiids and narrower than in *N. cecilialina.* The arrangement of the major cusps in USNM PAL V 11367 is sub-rhomboidal, contrasting with a more sub-rectangular arrangement displayed by *A. weltoni* and *P. tabatai*. *N. cecilialina* has a trapezoidal arrangement of its major cusps; however, little difference between left and right m2s has been found. In *N. cecilialina*, the entoconid-hypoconulid-extra cusps arrangement of the right side of m2 is straight, but the entoconid-hypoconulid-extra cusps arrangement of the left is bended to posterior side. The left side of hypoconulid is situated more posterior side than entoconid-extra cusps. In conclusion, based on the number and size of the major cusps, the arrangement of cusps, and the height of the extra cusp, we identify USNM PAL V 11367 as *Neoparadoxia* cf. *N. cecilialina*.

## Discussion

### Discovery and historiography of USNM PAL V 11367

With basic image enhancement tools (e.g., Adobe Photoshop), we were able to better resolve the original but faded specimen label in the collections associated with USNM PAL V 11367 (Fig. 1 and Related file 1). Specifically, we were able to make the now-faded handwritten notes legible (Fig. 1A,B), revealing critical information about the specimen. The widespread availability of image enhancement for faded fieldnotes and labels provides a new source of information for uncovering legacy issues in museum collections (e.g.^21–23^), especially in cases where locality data or collecting information cannot be well resolved.

Accession files with this specimen (Related file 1) show that it was gifted from Arthur M. Ames to the United States National Museum (now the National Museum of Natural History, Smithsonian Institution) on 15 October 1925, and approved by George P. Merrill, head curator of geology from 1917 to 1929. Prior to its accession to the museum, an anonymous individual identified the tooth as belonging to *Desmostylus hesperus*. Forty years later, on 17 November 1965, Charles A. Repenning reidentified this specimen as *Paleoparadoxia* sp. (Fig. 1A,B), an assertion that was incorporated into its catalog information. According to the label, USNM PAL V 11367 was collected in the city of Corona, Riverside County, California, yet no precise information of its geological provenance was recorded. On the backside of the label, there are notes (Fig. 1B) referring to the US Geologic Survey Corona South 7.5′ quadrangle map for Riverside and Orange counties, California^24^. However, no geographic location, exact horizon, nor lithology was stated, and the specimen’s collector, A. M. Ames, lived in Santa Barbara, California but died on 25 August 1939^21–23^.

In nearly a century after its discovery, the only mention of USNM PAL V 11367 was by Panofsky^25^, who listed it in a catalog of desmostylian tooth specimens used as a comparative basis for a mandible restoration of the “Stanford specimen” *N. repenningi*. Panofsky^25^ identified USNM PAL V 11367 as a left m2 with six main cusps, with no additional cusps (Table 1 in^25^), while also stating that this specimen has “an open lake in the center of each of the seven cusps” (^25^: p. 103). The inconsistency of this description differs from our own, which we attribute to differences in morphological criteria or a typographic error.

### Geological horizon and age of USNM PAL V 11367

In this paper, we refer to the “Topanga” Formation following recent studies^20,26,27^ of this geologic unit. This formation was originally based on a sequence of marine sandstones exposed in an anticline just west of Old Topanga Canyon in the central Santa Monica Mountains of Los Angeles County, California^28^. After its initial description, the name of the formation was applied to a much thicker and heterogeneous sequence of sedimentary and volcanic rocks^29^. Campbell et al.^30^ compiled the history and chronology of changes in usage of “Topanga” in the Miocene stratigraphic nomenclature in Southern California, showing that the criteria of continuous deposition and shared provenance were not demonstrated in every instance. Campbell et al.^30^ argued that strata assigned to the Topanga Formation in the Los Angeles Basin and eastern Ventura Basin areas are different from other units that have also been referred to the Topanga Formation in Orange County or in the Santa Monica Mountains of Los Angeles and Ventura counties. To distinguish these units, here we follow recent studies^20,26,27^ and use the name of “Topanga” Formation for the early to middle Miocene rocks bearing fossil marine mammals^20,26,31–33^ in Southern California.

According to the collections records (Fig. 1), USNM PAL V 11367 was collected in the city of Corona, Riverside County, California, USA. This city is in the western part of Riverside County, comprising an approximate area of 100 km^2^^34^. Previously, Panofsky^25^ suggested that USNM PAL V 11367 would have derived from the Temblor Formation (14.8 to 15.8 Ma^35^), likely as a guess based on the prevalence of desmostylian teeth recovered from this unit in central California, yet today there are no Temblor Formation outcrops mapped near Corona^24,36^; the closest Temblor outcrops are located in Fresno and Kern counties^37^, approximately 200 km away.

The geologic maps of Riverside County^24,36,38^ indicate that the city limits of Corona encompass a wide variety of sedimentary rocks from the Jurassic to the Holocene in age, but only a few marine deposits, such as the Jurassic Bedford Canyon Formation and the middle Miocene “Topanga” Formation are exposed^24,39^. Specifically, the marine sandstones of the “Topanga” Formation occur within the fault zone at the southeast and northwest of Corona.

Outside of Riverside County, the “Topanga” Formation has yielded a diverse assemblage of fossil marine vertebrates in Southern California^20,26,31^, including desmostylians referred to *Desmostylus hesperus* and *Paleoparadoxia* sp. in Orange County (Supplementary 1). USNM PAL V 11367 represents the second reported fossil marine mammal from Riverside County. Previously, an isolated record of “Cetacea indet.” was mentioned from the Zanclean stage Imperial Formation^40^ and Supplementary Data 2), which is exposed far east of Corona’s city limits.

In assessing the age of the “Topanga” Formation in Southern California, Boessenecker and Churchill^26,31^ argued that the land mammals (late Hemingfordian North American Land Mammal Age, represented by *Aepycamelus*, *Copemys* and *Merychippus*; 17.5–15.9 Ma^35,41^), benthic foraminifera, fossil mollusks, and K/Ar dating all placed the age range between 17.5 and 15 Ma for this geological unit^41^ in Orange County. More recently, Velez-Juarbe^20^ revised the age of “Topanga” Formation in this county to 16.5–14.5 Ma based on new foraminiferal zones presented in Ogg et al.^42^.

We propose that USNM PAL V 11367 derives from exposures of the “Topanga” Formation in Riverside County. If this mapped unit in Riverside can be correlated with “Topanga” Formation units in Orange County, it would imply a middle Miocene age, likely 16.5–14.5 Ma^20^, and given the morphological similarities of this isolated tooth with more complete paleoparadoxiid material in Orange County with stronger age constraints, we think a middle Miocene age for USNM PAL V 11367 is warranted. Given the reduced distribution of outcrops of the “Topanga” Formation^24,36^ in Corona, we identify two potential localities for USNM PAL V 11367 (Fig. 3). These two localities are situated in urbanized areas, less than 21 km apart, in the northwest and the southeast corners of Corona’s city limits (see Fig. 3B). Both are notably less than 40 km apart from the type locality of *N. cecilialina* in Orange County, but we urge skepticism for a direct correlation as the marine units of Riverside County requires detailed stratigraphic revision to determine their age constraints; they likely belong to a different depositional basin than “Topanga” Formation exposures in westward Southern California counties.

### Morphological variation and potential diversity of Paleoparadoxiidae

Our comparisons reveal considerable morphological variation in the arrangement and number of dental cusps across Paleoparadoxiidae (Fig. 4). The cusps arrangement for the m2-3 of *Archaeoparadoxia* and *Paleoparadoxia* were previously reported by Inuzuka et al.^43^ (Fig. 4B), but the addition of another specimen (USNM PAL V 11367) reveals larger morphological variability than previously known for the genus *Neoparadoxia* (Fig. 4C). Specifically, the holotype of *N. cecilialina* displays slightly different configurations between its right and left m2, driven mainly by the position of the hypoconulid in occlusal view (Fig. 4C). USNM PAL V 11367, the second known *Neoparadoxia* m2 (or the first m3), is comparable in size and shape with the same teeth in the type specimen of *N. cecilialina*, especially the right m2. Both the Smithsonian and LACM specimens display a horizontal alignment of the extra cusp, the hypoconulid, and the entoconid; nevertheless, USNM PAL V 11367 shows a tighter configuration, lacking a wide internal spacing between cusps characteristic of the type specimen of *N. cecilialina* (Fig. 4C). Given the known ontogenetic changes that affect the dental nomenclature in desmostylians^32,44^, the addition of more comparative material should help discriminate between competing statements of homology^45^. The identification of USNM PAL V 11367 from the “Topanga” Formation of Corona represents a second diagnostic record of *Neoparadoxia* from three separate Middle Miocene units in Southern California, reaffirming its presence as a Middle Miocene taxon: USNM PAL V 11367 from the “Topanga” Formation of Riverside County; *Neoparapdoxia* (LACM 6920) from the Altamira Shale^46^; *Neoparadoxia* from the Topanga Formation of Orange County^46,47^; and the holotype of *N. cecilialina* from the lower part of Monterey Formation in the Capistrano syncline, Orange County^46^. It is possible that other records of Palaeoparadoxiidae from Orange County (e.g.^47^) and elsewhere in California may represent *Neoparadoxia*. For example, Awalt et al.^32^ noted that a palaeoparadoxiid from Orange County identified by Panofsky as *Paleoparadoxia* sp. (LACM 131889)^25^ is better referred to Paleoparadoxidae sp., pending a more detailed evaluation of this material, which differs in clear ways from *N. ceciliana*. One of the benefits of continued descriptive work on desmostylian material from well-constrained stratigraphic contexts in Southern California will be the biostratigraphic opportunities for cross-basin comparisons, especially for exposures of the “Topanga” Formation.

Parham et al.^46^ emphasized that *Neoparadoxia* occurs widely in middle Miocene units across California: besides the aforementioned ones, Parham et al.^46^ noted records of this genus from the Sharktooth Hill Bonebed (LACM 120023), the Altamira Shale (LACM 6920), and the Ladera Sandstone^15^ (UCMP 81302). To date, *Neoparadoxia* is only known from California, yet it is likely that other paleoparadoxiid material tentatively assigned to other genera may expand the geographic range of this taxon. Interestingly, on the west side of the Pacific (Russia–Japan) and some parts of the east side of the Pacific (Oregon–Washington), *Desmostylus* spp. and paleoparadoxiids rarely co-occurred from the same formation^48,49^, yet there are many geological units in South California where desmostylids and paleoparadoxiids co-occurred (e.g., Santa Margarita Formation^50,51^, Rosarito Beach Formation^52^, Tortugas Formation^51^, and Temblor Formation^3,4^). The abundance of new material from the “Topanga” Formation from Orange and Riverside counties should contribute to the discussion of desmostylian environmental preferences^48,53^.

Lastly, like other marine mammal lineages, desmostylian body sizes reached their maximum body size late in their evolutionary history^54^. By the middle to late Miocene, desmostylians were the largest herbivorous marine mammals along the North Pacific coastlines^54^, although they likely competed ecologically with co-occurring sirenians, which later eclipsed desmostylians in body size and survived until historical times in the North Pacific Ocean^55^. Specifically, in the “Topanga” Formation of Orange County, desmostylians co-occurred with sirenians such as *Metaxytherium arctodites*^56^, an ecological association that likely was repeated elsewhere in the mid-Miocene of California (e.g., coeval deposits of the Round Mountain Silt). Given the improving stratigraphic picture of Southern California marine mammal-bearing localities, future work on desmostylian paleoecology could test hypotheses of competition with taxonomic co-occurrence data grounded in strong comparative taphonomic and sedimentological frameworks.

## Methods

We report on a single *Neoparadoxia* cf. *N. cecilialina* specimen from the “Topanga” Formation of Riverside County, California. This specimen was collected in 1913 without a precise record for its source locality by the collector, who died in 1939. The stratigraphic origin of USNM PAL V 11367 is confirmed by the museum records, which we explain in the Discussion section. USNM PAL V 11367 is permanently housed in the Department of Paleobiology collections at the National Museum of Natural History, Smithsonian Institution, Washington DC., the USA.

### Comparative specimens

We used some comparative specimens for USNM PAL V 11367. Comparative materials are listed in Table S1.

### Institutional abbreviations

LACM, Natural History Museum of Los Angeles County, Los Angeles, California, USA; NMNS, National Museum of Nature and Science, Tokyo, Japan; UCMP, University of California Museum of Paleontology, Berkeley, California, USA; USNM PAL, Department of Paleobiology, National Museum of Natural History, Smithsonian Institution, Washington, District of Columbia, USA.

## Supplementary Information

Supplementary Legends.

Supplementary Table S1.

Supplementary Information 3.

Supplementary Information 4.

Supplementary Information 5.

## Data Availability

All data generated during this study are included in this published article and its supplementary information files.
