# Supplementary material for: New data from the first discovered paleoparadoxiid (Desmostylia) specimen shed light into the morphological variation of the genus Neoparadoxia
Source: Sci Rep. 2022 Aug 21;12:14246. doi: 10.1038/s41598-022-18295-5 (PMC9393157; doi:10.1038/s41598-022-18295-5)
Supplement: Supplementary file 1 — Supplementary Legends. [file 41598_2022_18295_MOESM1_ESM.docx]

Table S1 The list of comparative specimens used in this study.

Related file 1.

Accession data for USNM PAL V 11367.

Supplementary Data 1.

Desmostylia occurrences from Topanga Formation in Orange County California, the USA. We downloaded this dataset from the Paleobiology Database on the 6^th^ of July, 2022.

Supplementary Data 2.

Mammalian fossil occurrences from Riverside County, California, the USA. We downloaded this dataset from the Paleobiology Database on the 22nd of August, 2021.
