# Supplementary material for: New data from the first discovered paleoparadoxiid (Desmostylia) specimen shed light into the morphological variation of the genus Neoparadoxia
Source: Sci Rep. 2022 Aug 21;12:14246. doi: 10.1038/s41598-022-18295-5 (PMC9393157; doi:10.1038/s41598-022-18295-5)
Supplement: Supplementary file 3 — Supplementary Information 3. [file 41598_2022_18295_MOESM3_ESM.pdf]

Smithsonian Institution Archives

USNM Accession Records: 1926 Acc. No. 89024

This document is provided by the Smithsonian Institution Archives. We welcome you to use it for personal and educational uses. For commercial uses, please contact osiaref@si.edu.

Please cite as –

Short citation:

Smithsonian Institution Archives, Record Unit 305, SIA000305_R338_Y1926_A0089024

Long citation:

Smithsonian Institution Archives, Record Unit 305, United States National Museum, "USNM Accession Records: 1926 Acc. No. 89024", SIA000305_R338_Y1926_A0089024

When citing our collections online, please link to the Smithsonian Institution Archives http://siarchives.si.edu.

ACCESSION No. 89024

OFFICE OF THE REGISTRAR
SMITHSONIAN INSTITUTION

ARTHUR M. AMES

ACCESSION 89024 **Cat. No.** 11,367

OCT 21 1925

SMITHSONIAN INSTITUTION
UNITED STATES NATIONAL MUSEUM

ACCESSION MEMORANDUM

Department of Geology *Division of* Vert. Paleont.

October 15, 1925, *192*

Please enter as an accession from Arthur M. Ames.

(Address) 1910 Cleveland Avenue.

Santa Barbara, California.

the following object (collected ~~with~~ *without the aid of a Museum outfit):*

A tooth of Desmostylus hesperus Marsh, from Riverside County, California.

Papers appended memorandum

(The Administrative Assistant directs that ALL letters in the possession of the Curator or his Assistants, which relate to this Accession, be attached to this memorandum and forwarded with it to the Division of Correspondence.)

Gift, ~~exchange, loan, deposit, transfer, bequest, collected for the Museum, purchased, made in the Museum~~

Geo. P. Merrill

m Head *Curator.*

DEPT. OF GEOLOGY OCT 2[illegible] 1925 GEORGE P. MERRILL

13—106

ENTERED

ACCESSION. 89024

Oct. 14. 1924

Miss Moodey:

Will you accession as a gift from Mr Arthur M. Ames, 1910 Cleveland Ave, Santa Barbara, California. one tooth of Desmostylus hesperus Marsh.

The tooth was presented in person and is from near Corona, Riverside County, California.

C.W. Gilmore

ACCESSION 89024

October 19, 192[illegible].

Mr. Arthur M. Ames,
1910 Cleveland Avenue,
Santa Barbara, California.

Dear Sir:

I beg to acknowledge the receipt of a tooth of Desmostylus hesperus Marsh, from Riverside County, California, which you have kindly presented to the National Museum. It is entered on the records as a gift in your name, and I would assure you of my thanks.

Very truly yours,

A. WETMORE

Sm

Assistant Secretary.
